# Development of highly sensitive metal-ion chemosensor and key-lock anticounterfeiting technology based on oxazolidine

**DOI:** 10.1038/s41598-022-05098-x

**Published:** 2022-01-20

**Authors:** Bahareh Razavi, Hossein Roghani-Mamaqani, Mehdi Salami-Kalajahi

**Affiliations:** 1 Faculty of Polymer Engineering, Sahand University of Technology, P.O. Box 51335-1996, Tabriz, Iran; 2 Institute of Polymeric Materials, Sahand University of Technology, P.O. Box 51335-1996, Tabriz, Iran

**Keywords:** Materials for optics, Structural materials, Natural hazards

## Abstract

Optical chemosensors and ionochromic cellulosic papers based on oxazolidine chromophores were developed for selective photosensing of metal ions and information encryption as security tags, respectively. The oxazolidine molecules have been displayed highly intense fluorescent emission and coloration characteristics that are usable in sensing and anticounterfeiting applications. Obtained results indicated that oxazolidine molecules can be used for selective detection of pb^2+^ (0.01 M), and photosensing of Fe^3+^, Co^2+^ and Ag^+^ metal ion solutions by colorimetric and fluorometric mechanisms with higher intensity and sensitivity. Also, oxazolidine derivatives were coated on cellulosic papers via layer-by-layer method to prepare ionochromic papers. Prepared ionochromic papers were used for printing and handwriting of optical security tags by using of metal ion solutions as a new class of anticounterfeiting inks with dual-mode fluorometric and colorimetric securities. The ionochromic cellulosic papers can be used for photodetection of metal ions in a fast and facile manner that presence of metal ions is detectable by naked eyes. Also, key-lock anticounterfeiting technology based on ionochromic papers and metal ion solution as ink is the most significant strategy for encryption of information to optical tags with higher security.

## Introduction

Timely detection of different analytes by using chemo- or bio-sensors could be increased the safety level and inhibits environmental hazards^1–6^. Among various methods, optical sensing is the most efficient approach for detection of environmental changes with high accuracy and validity^7–14^. In the recent years, photosensing of metal ions in biological environments has been considered in advanced researches. The presence of toxic ions, even in low concentrations, could lead to irreparable damage to human life. Stimuli-responsive materials have remarkably been used to design advanced sensors with the ability of monitoring various triggers depending on their chemical structure and functional photoactive molecules^11,15–23^. Therefore, a wide range of responses including color change, solubility, deformation, volume variation, and induction of surface charge could be observed as an alarm to display detection of the stimuli. Optical sensors could be used for detection of external stimuli, which are classified into chemical, physical, and biochemical categories^24–31^. Fluorescent compounds as light-responsive materials are among the most important smart systems, which have been used in preparation of optical sensors. Fluorescent compounds change their optical properties, such as color and fluorescence emission, in response to external triggers, which are detectable by UV–vis and fluorescence spectroscopies^16,32–36^.

Oxazolidine as a novel stimuli-chromic compound have significant advantages including fast responsivity towards external stimuli, isomerization between two isomer structures as non-fluorescent/discolored and colored/fluorescent states^37–47^. In addition, a wide range of oxazolidine derivatives could be synthesized by using various aromatic aldehyde compounds substituted with different electron donor and electron withdrawing groups. Oxazolidine derivatives display various stimuli-chromism, such as hydrochromism, solvatochromism, acidochromism, and photochromism, depending on the functional groups on their chemical structure^45,47–50^. Oxazolidine displays various coloration and fluorescence emission in different media, depending on interactions of different functional groups on the indolenine ring or benzaldehyde structure with the surrounding media^45,51–55^. The color changes act as an alarm in response to stimulus that is detectable by naked eye, which is a significant requirement for development of highly selective colorimetric and fluorometric sensors without the need to high-cost materials or special equipment. These unique properties have resulted in introduction of oxazolidine as a good candidate for chemosensor applications in the recent years.

Herein, new fluorescent oxazolidine derivatives were synthesized by using different aromatic aldehydes, such as 4-nitrobenzaldehyde and 9-anthraldehyde. The functional groups (electron donating or electron withdrawing) could induce various responsivities to oxazolidine molecules, such as coloration or fluorescence emission in presence of metal ions. Because of the pointed advantages, oxazolidine derivatives could be used as an excellent optical chemosensor for determination of various metal cations with high sensitivity and selectivity. The substituted functional groups such as NO_2_ and anthracene are able to coordinate with metal ions via non-bonding electrons or π-π interactions, which could result in coloration or fluorescence emission. Therefore, optical properties of the oxazolidine derivatives upon various metal ions were studied by UV–vis and fluorescence spectroscopies to develop novel molecular colorimetric/fluorometric chemosensors. The oxazolidine derivatives were synthesized for the first time and also used as a novel type of colorimetric/fluorometric optical chemosensors for selective and sensitive detection of metal ions. In addition, oxazolidine derivatives have potential applications as paper-based ionochromic chemosensors for detection of metal ion solutions.

## Experimental

### Materials

2,3,3-Trimethylindolenin (98%), 1,1,2-trimethylbenz[e]indole (98%), 2-bromoethanol (95%), 9-anthraldehyde, and 4-nitrobenzaldehyde (98%), which were used for the synthesis of (E)-1-(2-hydroxyethyl)-3,3-dimethyl-2-(4-nitrostyryl)-3H-indol-1-ium (OX_1_-Nitro), (E)-3-(2-hydroxyethyl)-1,1-dimethyl-2-(4-nitrostyryl)-1H-benzo[e]indol-3-ium (OX_2_-Nitro), (E)-2-(2-(anthracen-9-yl)vinyl)-1-(2-hydroxyethyl)-3,3-dimethyl-3H-indol-1-ium (OX_1_-Anthracene), and (E)-2-(2-(anthracen-9-yl)vinyl)-3-(2-hydroxyethyl)-1,1-dimethyl-1H-benzo[e]indol-3-ium (OX_2_-Anthracene) were purchased from Sigma-Aldrich. The metal salts including Zn(NO_3_)_2_, Cu(NO_3_)_2_, Ni(NO_3_)_2_, Co(NO_3_)_2_, Pb(NO_3_)_2_, Fe(NO_3_)_3_, Cd(NO_3_)_2_, AgNO_3_, and Hg(NO_3_)_2_ were supplied by the Merck Chemical Company and used for the preparation of aqueous solutions of metal ions. Distilled-deionized (DI) water was used in all the recipes, and all the materials were used without further purifications.

### Synthesis of the oxazolidine derivatives

**Figure 1:**
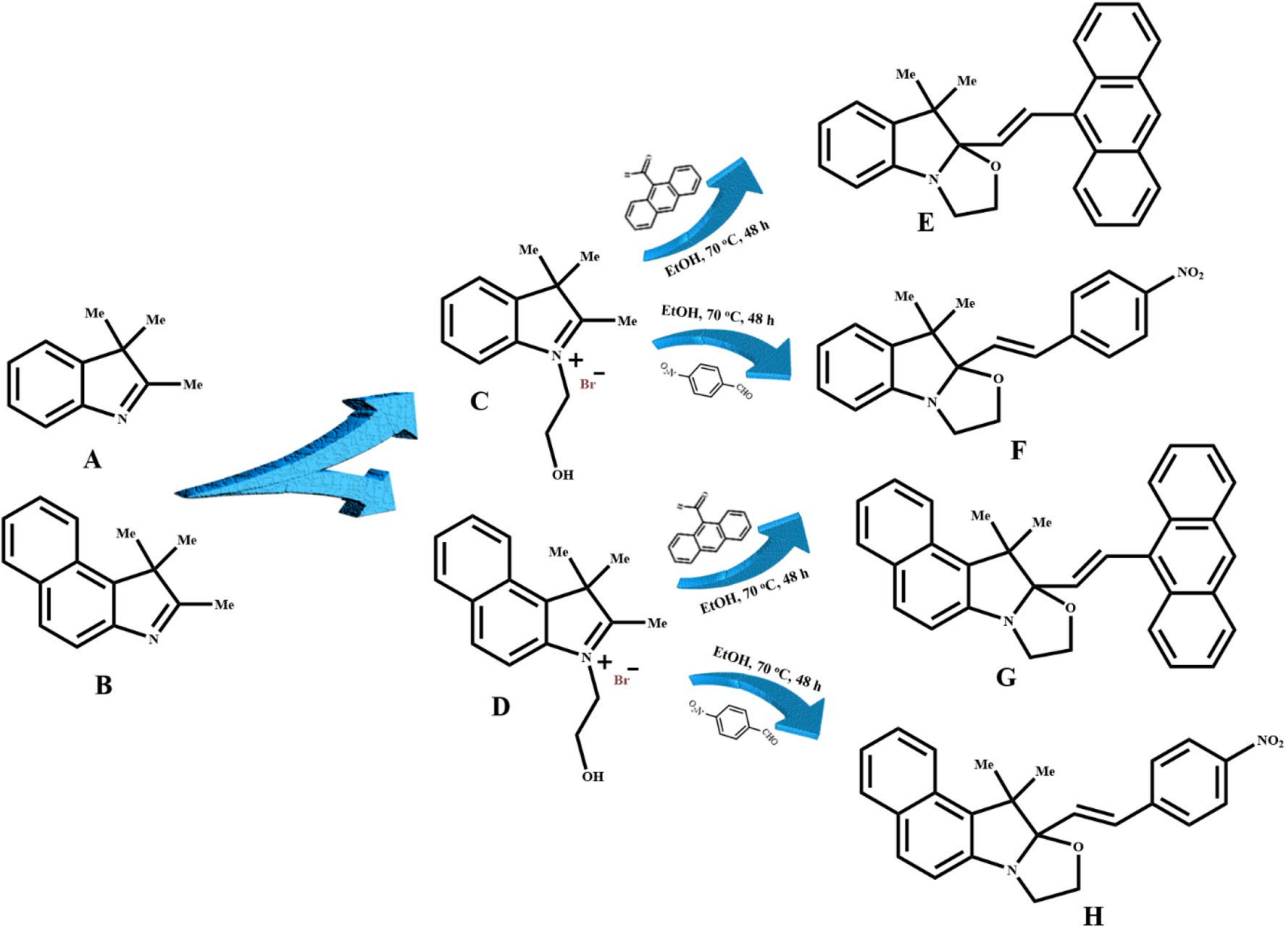
Schematic representation for the synthesis of different oxazolidine derivatives from various indolium salts.

The oxazolidine derivatives were synthesized according to the literature^40,41,44,47,56^. A solution of 2-bromoethanol (25 mmol, 1.8 mL) and 2-buotanone (10 mL) was added dropwise to the solution of 2,3,3-trimethylindolenin (A, 20 mmol, 3.2 g) or 1,1,2-trimethylbenz[e]indole (B, 20 mmol, 2.1 g) and 2-buotanone (30 mL) at ambient conditions under reflux at 80 °C for 48 h to obtain reddish precipitates. The precipitate (C or D, respectively, Fig. 1) was washed three times with acetone to afford reddish solids with a reaction yield of about 60–70%. In the second step, 10 mmol of C (2.85) or D (3.35 g) was dissolved in ethanol (20 mL), and then a solution of 4-nitrobenzaldehyde (20 mmol, 2.45 g) or 9-anthraldehyde (20 mmol, 2.45 g) in 10 mL ethanol was added to the mixture and the reaction was continued for 48 h. The products were filtered and washed with cold ethanol (5 mL) for three times to obtain E (OX_1_-Anthracene), F (OX_1_-Nitro), G (OX_2_-Anthracene), and H (OX_2_-Nitro) as oxazolidine derivatives. Chemical structures of OX_1_ and OX_2_ were characterized by ^1^H NMR analysis (CDCl_3_ as solvent) and the corresponding spectra are presented in Fig. S1 (A, B, C, and D, Supporting Information) for the OX_1_-Anthracene, OX_1_-Nitro, OX_2_-Anthracene, and OX_2_-Nitro compounds, respectively.

### Preparation of the ionochromic security papers

The ionochromic security papers were prepared by a layer-by-layer method according to the procedure reported by Sheng et al.^41^. Whatman filter papers (Whatman qualitative filter paper for technical use, product number: WHA10347512, creped circles with a diameter of 185 mm, Germany) as the substrate were coated with a thin layer of 10 wt% PEG aqueous solution by impregnation and then drying at room temperature. Then, a solution of oxazolidine (0.08 mmol/L) in ethanol/H_2_O (2/3 by volume) containing 6 wt% of PEG and 0.01 wt% of NaNO_3_ was coated on the PEG-coated papere as the second layer. Finally, a layer of 10 wt% PEG solution was coated as the third layer on the two-layer coated papers to prepare the ionochromic security papers.

### Characterization

Chemical structure of the oxazolidine derivatives was characterized by proton nuclear magnetic resonance (^1^H NMR) spectroscopy using a Bruker DPX 400 MHz apparatus in D_2_O solvent. Optical properties of the samples were investigated by UV–vis analysis by using Jenway 6705 UV/Visible Scanning Spectrophotometer (United Kingdom). Fluorescence emission of the samples was studied by using JASCO FP-750 Spectrofluorometer (Japan). Diluted samples with a concentration of 0.5 mg/mL were used for UV–vis and fluorescence spectroscopy analysis. To evaluate the fluorescence properties, excitation was done by a UV lamp (365 nm, 50 W/m^2^), CAMAG 12VDC/VAC (50/60 Hz, 14 VA, Switzerland). Also, the source for visible light was a common LED lamp (8 W/m^2^).

## Results and discussion

The photochromic and fluorescent compounds well-known as stimuli-chromic materials have extensively been used in development of the colorimetric and fluorometric chemosensors in the recent years^12,29,57–60^. Oxazolidine is a new category of stimuli-chromic materials that displays hydrochromism, acidochromism, photochromism, and ionochromism by induction of different external stimuli. Compared to the other stimuli-chromic materials, chemical structure of the oxazolidine derivatives is very sensitive to characteristics of the surrounding media, where coloration and fluorescence emission could be significantly affected by the solute–solvent interactions. Therefore, the oxazolidine molecules are highly important candidates for colorimetric and fluorometric photosensing of different analytes especially metal ions^11,44,52,61,62^. Chemical structure and substituted functional groups on oxazolidine molecules are significant parameters on sensitivity and selectivity of metal ion chemosensors. In this study, some novel oxazolidine derivatives were synthesized according to the procedure schematically presented in Fig. 1 by using various aromatic aldehydes. OX_1_-Anthracene and OX_2_-Anthracene derivatives were prepared with two indolenine salts and 9-anthraldehyde, and OX_1_-Nitro and OX_2_-Nitro were synthesized by using the same indolenine salts and 4-nitrobenzaldehyde. Chemical structure of the oxazolidine derivatives were characterized by ^1^H NMR analysis and the obtained spectra were reported in Fig. S1. All of the protons were indicated by the labels in chemical structure of the oxazolidine derivatives, and the corresponding peaks were determined in the spectra shown in Fig. S1. Results displayed successful synthesis of the oxazolidine derivatives that were used as colorimetric/fluorometric sensors for photodetection of metal ions.

### Photodetection of metal ions by oxazolidine derivatives

**Figure 2:**
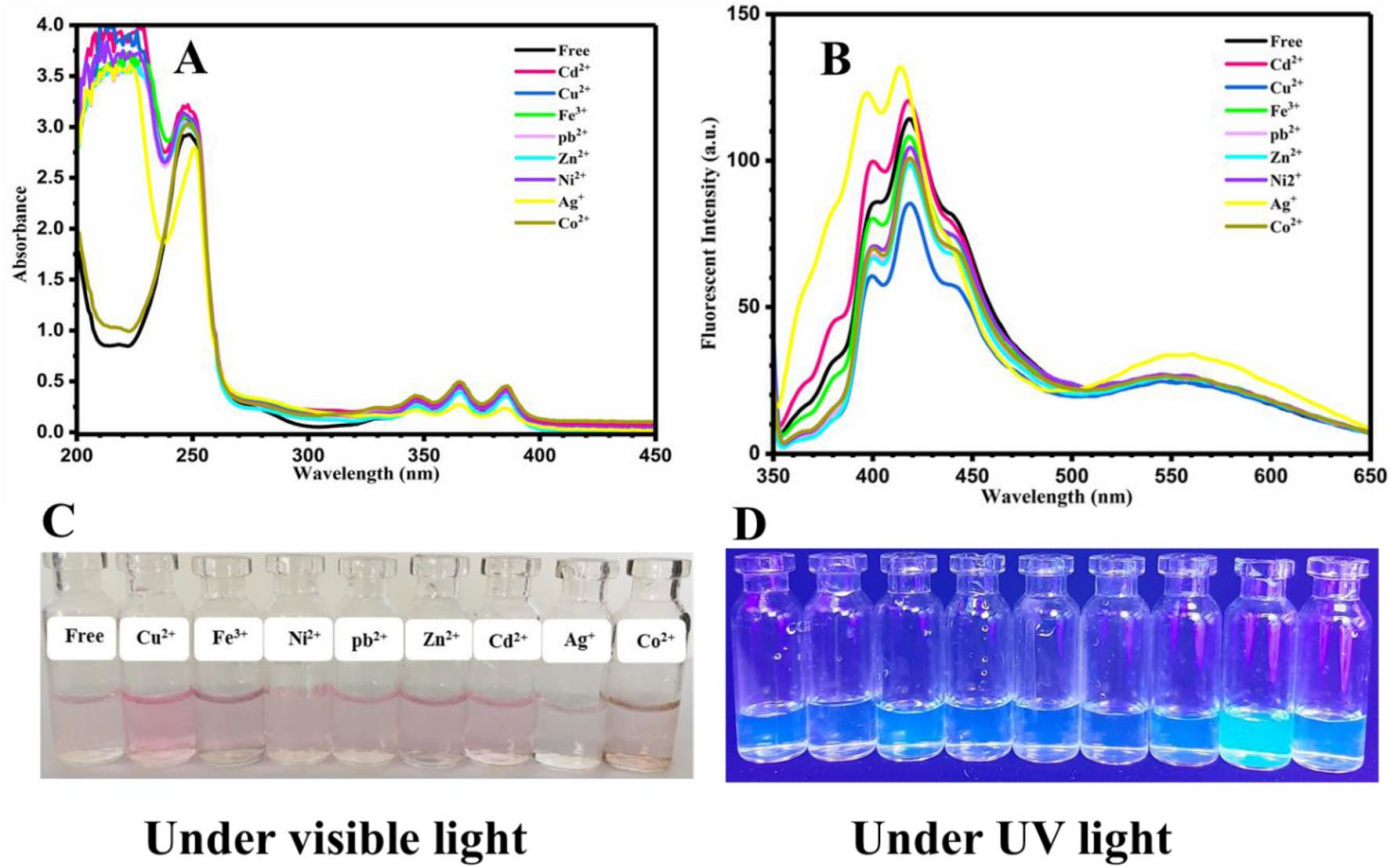
(**A**) UV–vis and (**B**) fluorescence spectra (excited with wavelength of 340 nm) of the OX_1_-Anthracene aqueous solutions with a concentration of 0.0002 M containing metal ions with a concentration of 0.01 M, and photographs of (**C**) color change and (**D**) fluorescence emission for the OX_1_-Anthracene/metal ions solutions under visible light and UV irradiation, respectively.

Metal ions even in low amount, especially in living media, could have irreparable damages in daily life. Efficient detection of metal ions could be carried out by optical chemosensors using organic compounds with the ability of colorimetric and fluorometric response to photo irradiation. For this purpose, aqueous solutions of oxazolidine derivatives (0.0002 M) were used for photodetection of metal ions (0.01 M) including Cd^2+^, Cu^2+^, Fe^3+^, Pb^2+^, Zn^2+^, Ni^2+^, Co^2+^, and Ag^+^ via UV–vis and fluorescence spectroscopies. As shown in Fig. 2, photodetection of metal ions in OX_1_-Anthracene solution was investigated by UV–vis (Fig. 2A) and fluorescence spectroscopy (Fig. 2B). In UV–vis spectra, a broad peak in the range of 220–300 nm with a *λ*_*max*_ of about 245 nm and also multiple broad peaks in the range of 330–400 nm were observed for OX_1_-Anthracene. Addition of various metal ions to the OX_1_-Anthracene solution was led to slight decrease of absorbance intensity for both of the peaks (220–300 and 330–400 nm), and showed that the OX_1_-Anthracene solution behaves selective for the detection of Ag^+^. Figure 2B displays fluorescence spectra of the OX_1_-Anthracene solutions containing different metal ions. All of the samples show similar fluorescence emission spectra, except the sample containing Ag^+^, which shows a shift in the peak point of fluorescence spectrum and increase of intensity. The photographs of colorimetric and fluorometric detection of Ag^+^ are presented in Fig. 2C, D, respectively, which show changing coloration from light-pink in free solution to discolored state for Ag^+^ solution. These results indicated that OX_1_-Anthracene could be used as a selective colorimetric/fluorometric optical chemosensor for detection of Ag^+^ by spectroscopy methods and even by naked eye.

**Figure 3:**
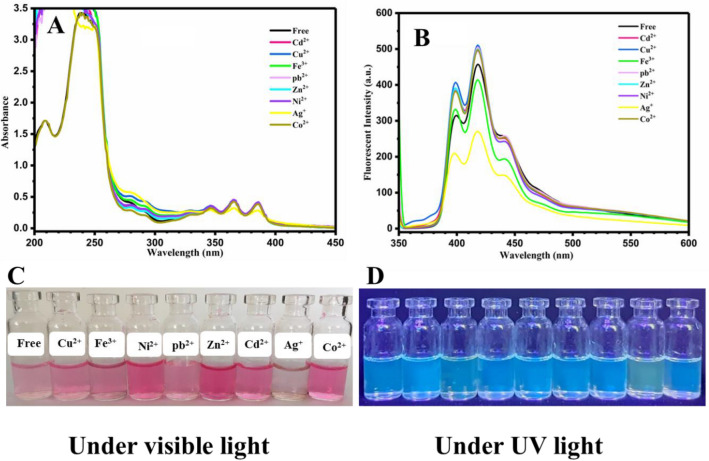
(**A**) UV–vis and (**B**) fluorescence spectra (excited with wavelength of 340 nm) of the OX_2_-Anthracene aqueous solutions with a concentration of 0.0002 M containing metal ions with a concentration of 0.01 M, and photographs of (**C**) color change and (**D**) fluorescence emission of the OX_2_-Anthracene/metal ions solutions under visible light and UV irradiation, respectively.

A significant effective parameter on the optical properties of the oxazolidine derivatives is the conjugation length in chemical structure. Substitution with electron withdrawing or electron donating groups can also significantly affect the sensing characteristics. UV–vis and fluorescence spectra of the OX_2_-Anthracene in aqueous solutions of various metal ions are shown in Fig. 3A, B, respectively. A significant reduction of absorption intensity was observed for the OX_2_-Anthracene sample after addition of Ag^+^, in comparing with the other metal ions. A remarkable decrease of fluorescence intensity was also observed for the OX_2_-Anthracene sample containing Ag^+^, which is important for photosensing of Ag^+^ by fluorometric method. Figure 3C, D were obtained from exposing the OX_2_-Anthracene/metal ions solutions to visible light (to observe coloration) and UV light irradiation (to observe fluorescence emission), respectively. All of the samples showed pink and light pink colors; however, the sample containing Ag^+^ was discolored. A light blue fluorescence emission was observed for all of the samples except the sample containing Ag^+^ that displayed light green fluorescence emission. The results indicate that OX_2_-Anthracene could be used as a highly-selective colorimetric/fluorometric optical chemosensor for photosensing of Ag^+^ by spectroscopy methods, and also easy detection by naked eye under visible light and UV irradiation by means of color change or fluorescence emission change after addition of Ag^+^.

**Figure 4:**
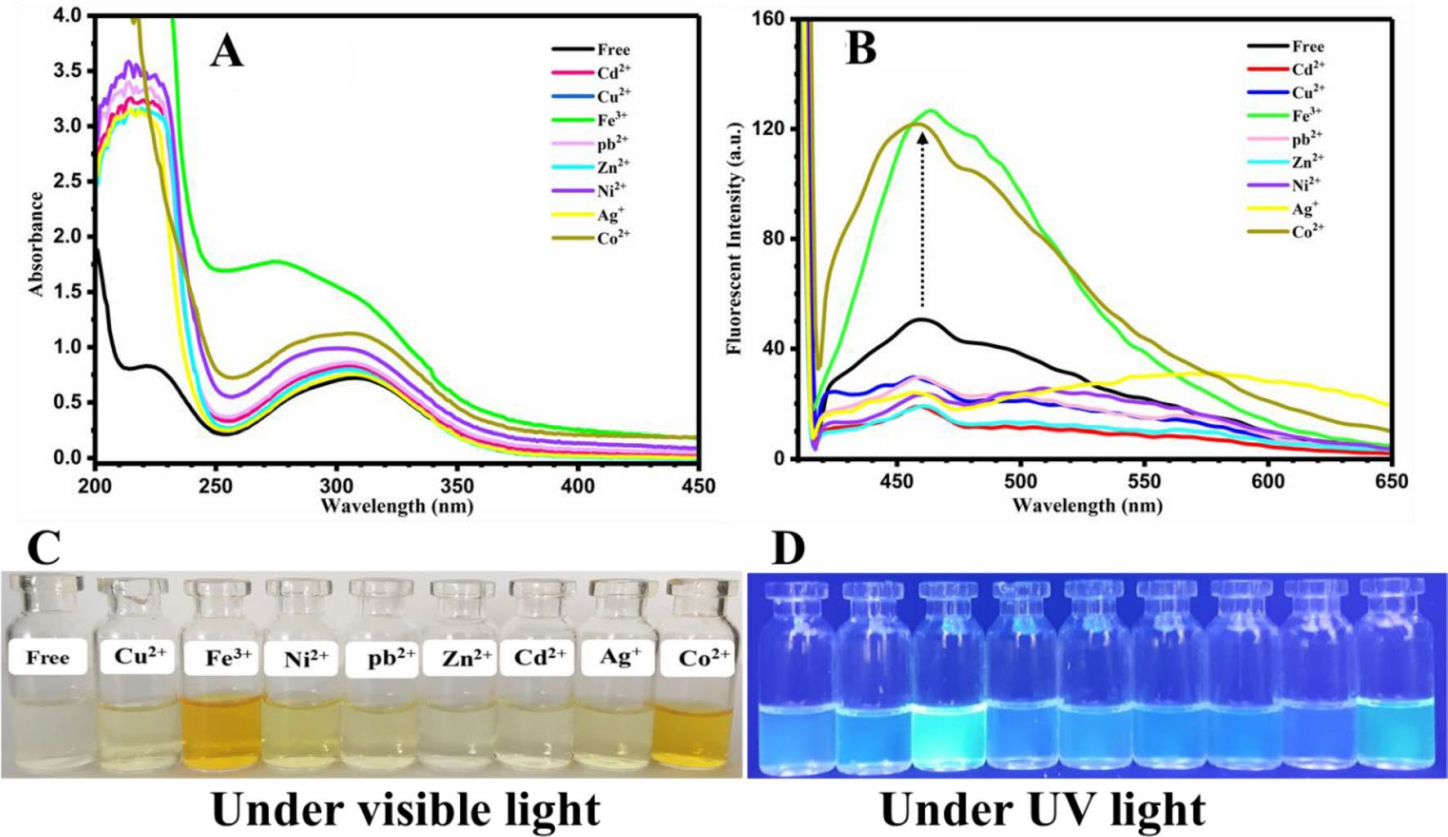
(**A**) UV–vis and (**B**) fluorescence spectra (excited with wavelength of 400 nm) of the OX_1_-Nitro aqueous solutions with a concentration of 0.0002 M containing metal ions with a concentration of 0.01 M, and photographs of (**C**) color change and (**D**) fluorescence emission of the OX_1_-Nitro/metal ions solutions under visible light and UV irradiation, respectively.

According to the important role of different functional groups on photochromism and fluorescence properties, two new oxazolidine derivatives of OX_1_-Nitro and OX_2_-Nitro were synthesized using 4- nitrobenzaldehyde as the raw material. The UV absorption (Fig. 4A) indicated that OX_1_-Nitro has a broad peak in 250–400 nm with *λ*_*max*_ of 310 nm. After addition of metal ions to the OX_1_-Nitro solution, hyperchromism phenomenon was observed by increasing of absorbance intensity for all of the samples, which is maximum in the case of Fe^3+^ and Co^2+^ metal ions. As shown in Fig. 4B, fluorescence intensity (excitation wavelength of 400 nm) in the range of 410–600 nm was increased in the case of Co^2+^ and Fe^3+^ solutions. These significant results, highly intense fluorescence emission, indicate high sensitivity and selectivity of OX_1_-Nitro toward Co^2+^ and Fe^3+^ metal ions. Coloration and fluorescence emission of the samples were investigated under visible light and UV irradiation, and the images are presented in Fig. 4C, D, respectively. As concluded from the results of UV–vis and fluorescence spectra, OX_1_-Nitro displayed highly-intense color change from discolored to bold yellow under visible light irradiation and also cyan blue fluorescence emission under UV irradiation after the addition of Fe^3+^ and Co^2+^ metal ions. Therefore, OX_1_-Nitro behaved as a selective and sensitive dual mode colorimetric/fluorometric optical sensor for photodetection of Fe^3+^ and Co^2+^ under visible or UV light irradiation.

**Figure 5:**
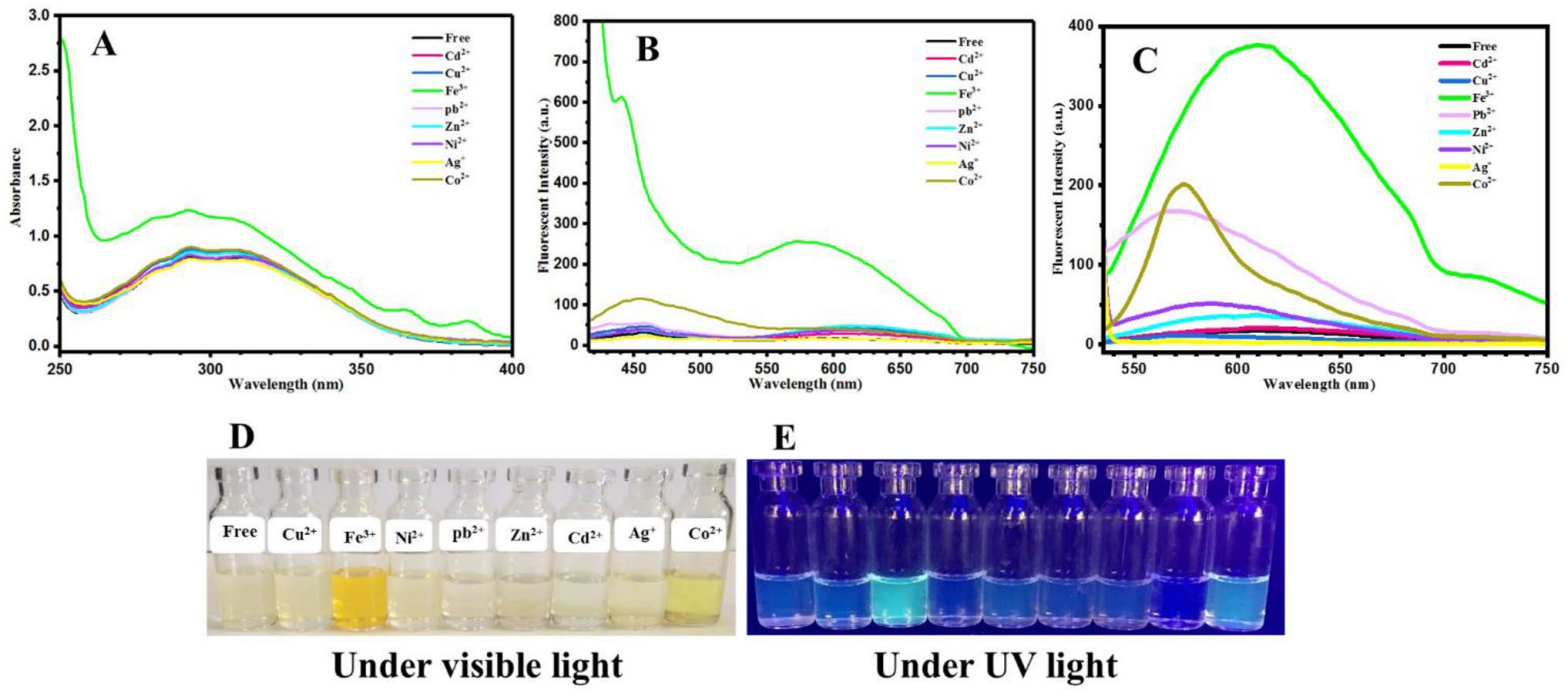
(**A**) UV–vis and (**B**, **C**) fluorescence spectra (excited at 400 and 520 nm, respectively) of the OX_2_-Nitro aqueous solutions with a concentration of 0.0002 M containing metal ions with a concentration of 0.01 M, and photographs of (**D**) color change and (**E**) fluorescence emission of the OX_2_-Nitro/metal ions solutions under visible light and UV irradiation, respectively.

To investigate effect of conjugation length of the oxazolidine structure on the photochromic and fluorescence properties, OX_2_-Nitro was synthesized by using a new indolenine salt. As shown in Fig. 5A, a broad absorbance peak in the range of 250–400 nm with *λ*_*max*_ of 310 nm was observed that is attributed to the absorption of UV light by OX_2_-Nitro (free sample, black curve). After addition of Fe^3+^, a bathochromism (red-shift of the peak wavelength) phenomenon occurred in addition to the hyperchromism (increasing of absorbance intensity) in UV–vis spectra (Fig. 5A). A highly intense and selective detection of Fe^3+^ by fluorescence emission was observed in fluorescence spectra (Fig. 5B), where the samples were excited by UV with the wavelength of 400 nm. The OX_2_-Nitro sample displayed emission by excitation with 520 nm in addition to the excitation with 400 nm, which could be named as a two-photon excitable fluorescent compound. Therefore, emission of the OX_2_-Nitro samples was investigated under excitation at 520 nm, and the resulted fluorescence spectra are presented in Fig. 5C. Surprisingly, a strong fluorescence emission was observed for the OX_2_-Nitro in response to the addition of Fe^3+^, Co^2+^, and also Pb^2+^ metal ions. Selective photosensing of Pb^2+^ in aqueous solutions, especially in living media, is a very significant challenge. It was shown that OX_2_-Nitro is a good candidate for development of a selective and sensitive chemosensor for detection of this toxic metal ion. In addition, the images obtained from photography under visible light (Fig. 5D) showed that the bold yellow color of OX_2_-Nitro containing Fe^3+^ and Co^2+^ was changed to highly intense cyan blue emission under UV light irradiation (Fig. 5E). In summary, OX_2_-Nitro can be used as a highly selective and sensitive optical chemosensor for simultaneous detection of Fe^3+^, Co^2+^, and Pb^2+^ metal ions by the two-photon excitable fluorometric approach and also colorimetric method, as spectroscopic and easy detection by naked eyes, respectively.

### Ionochromic security papers

**Figure 6:**
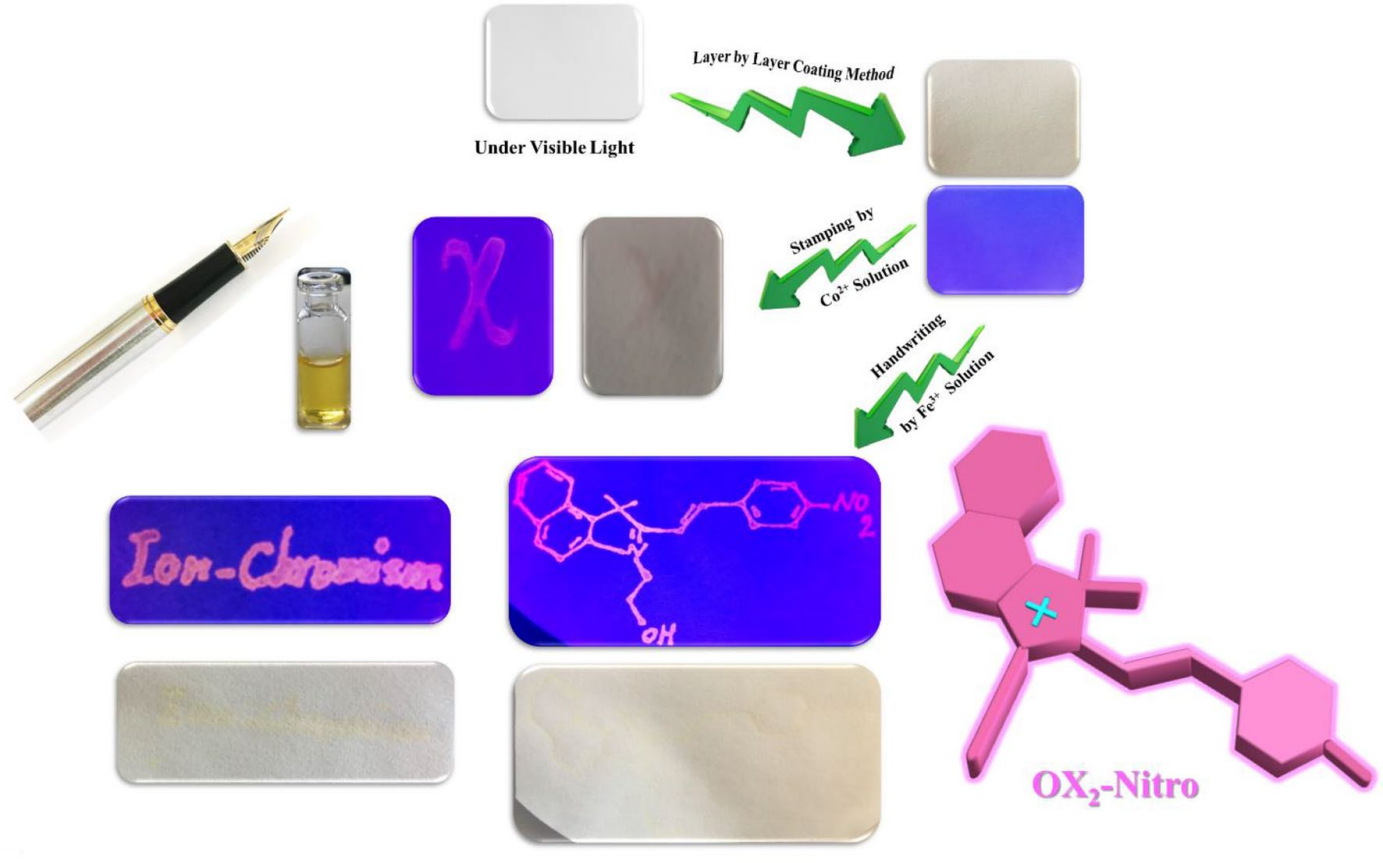
Handwriting and stamping of Fe^3+^ and Co^2+^ aqueous solutions on the ionochromic security papers coated with OX_2_-Nitro.

Anticounterfeiting technology has attached remarkable attentions in the preparation of smart inks, papers, fibers, labels, holograms, etc*.*^63–70^. Due to the important role of anticounterfeiting security papers in the recent studies, development of the anticounterfeiting ionochromic papers based on oxazolidine derivatives can help the documents to stay safe from any forgery and counterfeiting. The previous results showed that the fluorescent oxazolidine can be used as an excellent sensitive chemosensor for detection of metal ions with high selectivity and also for development of security papers for printing and handwriting by using of metal ion aqueous solutions as the ink. According to the selective photodetection of Fe^3+^ and Co^2+^ ions by OX_1_-Nitro and OX_2_-Nitro and Ag^+^ ion by OX_1_-Anthracene and OX_2_-Anthracene in this study, the ionochromic security papers were designed and prepared with specific inks based on metal ion aqueous solutions for handwriting and printing. For this purpose, the layer-by-layer technique was used to prepare the ionochromic security papers. A novel approach was developed in the current study for the first time to prepare new class of security papers with specific anticounterfeiting inks. As shown in Fig. 6, the ionochromic cellulosic paper was colorless under visible light, and displayed blue emission under UV irradiation (365 nm) because of its coating with oxazolidine solution (OX_2_-Nitro) by the layer-by-layer method. The Fe^3+^ and Co^2+^ metal ion aqueous solutions can induce highly intense cyan blue fluorescence emission when used as the ink for handwriting and printing (stamping) on the ionochromic papers coated with OX_2_-Nitro (Fig. 6). The highly intense pink fluorescence emission resulted from stamping and handwriting of Fe^3+^ and Co^2+^ metal ion aqueous solutions on security papers displayed dual-mode security based on simultaneously coloration and fluorescence emission. A significant and interesting application for the ionochromic papers is development of paper-based chemosensors for detection of metal ions under UV irradiation, similar to the paper-based indicators used for detection of pH in aqueous solutions.

**Figure 7:**
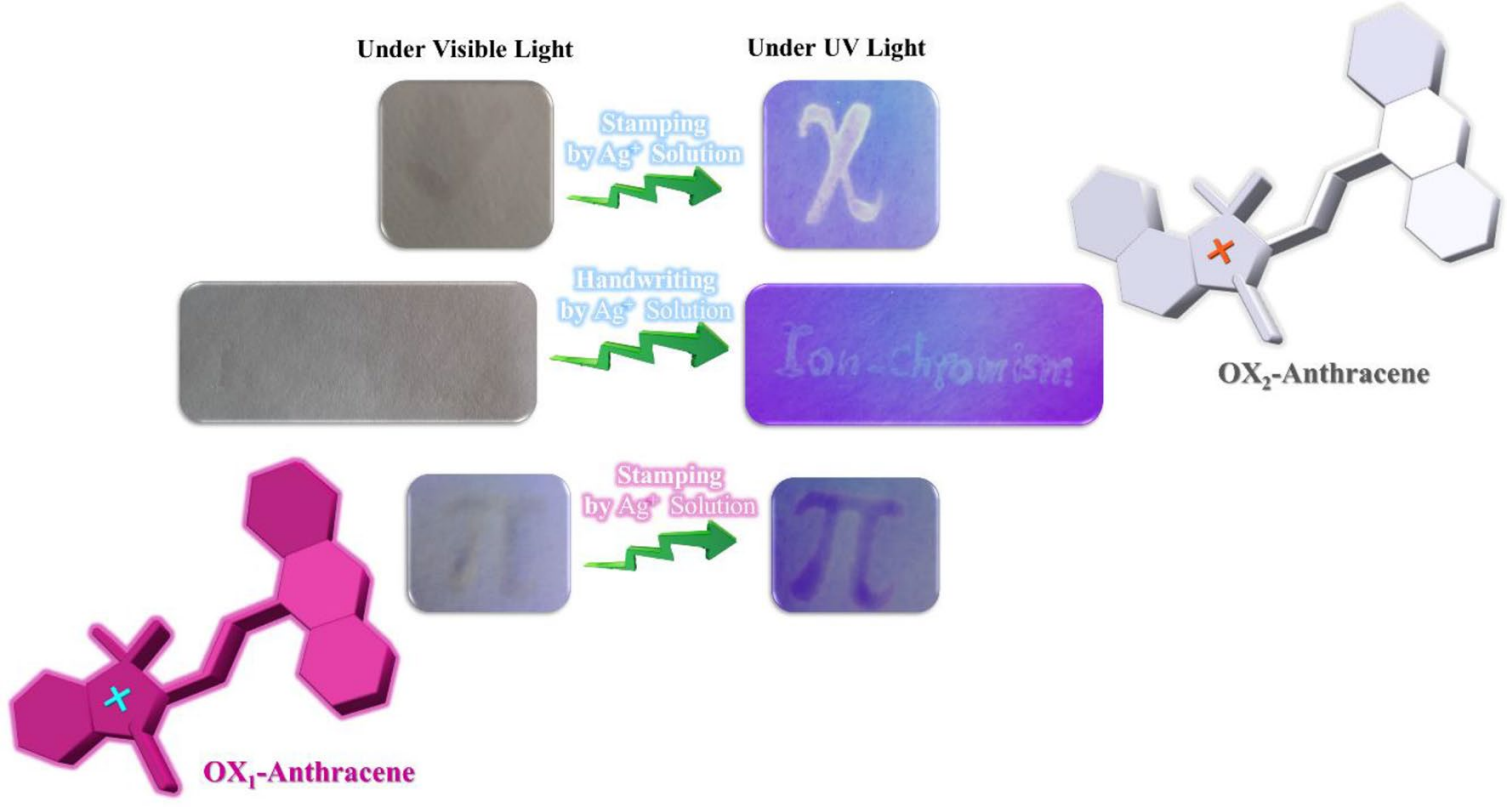
Handwriting and stamping of Ag^+^ aqueous solutions on the ionochromic security papers coated with OX_1_-Anthtacene and OX_2_-Anthtacene.

Similar ionochromic security papers were prepared by using OX_1_-Anthracene and OX_2_-Anthracene solutions coated on cellulosic papers by the layer-by-layer strategy. As previously discussed in Figs. 2 and 3, addition of the Ag^+^ aqueous solution into the OX_1_-Anthracene and OX_2_-Anthracene solutions was led to induction of highly intense cyan blue emission under UV light, which is a powerful approach for development of a novel anticounterfeiting technology. As shown in Fig. 7, the withe and dark purple security marks were induced on the papers coated with OX_1_-Anthracene and OX_2_-Anthracene by printing and handwriting of the Ag^+^ aqueous solution as an ionochromic security ink. The colorimetric/fluorometric properties of these novel ionochromic papers have a great powerful identifier for security documents based on ion metal solutions and also paper-based chemosensors for detection of metal ions under UV irradiation.

## Conclusion

Four novel oxazolidine derivatives with dual colorimetric and fluorometric characteristics were synthesized for selective photodetection of different metal ions with high sensitivity. The results showed that the OX_1_-Anthracene and OX_2_-Anthracene derivatives are able to selective photodetection of Ag^+^ in aqueous solution, and both of the OX_1_-Nitro and OX_2_-Nitro aqueous solutions can be used for selective photodetection of Fe^3+^ and Co^2 +^ metal ions. The detection in all of the samples is based on the fluorometric and colorimetric mechanisms, which can be observed with high intensity by naked eyes. The photodetection and also two-photon emission of OX_2_-Nitro could be used for selective detection of toxic Pb^2+^ (0.01 M) in aqueous solutions. As an innovative approach, such unique colorimetric and fluorometric properties of the oxazolidine derivatives after the addition of metal ions have potential applications in high security ionochromic papers for printing and handwriting of metal ion aqueous solutions as anticounterfeiting inks. The dual-mode colorimetric/fluorometric behavior of the ionochromic paper could be used for development of paper-based chemosensors for photodetection of Fe^3+^, Co^2+^, and Ag^+^ metal ion solutions. These observations could open a new window for development of high-tech chemosensors in security documents with easy and accurate detection of metal ion aqueous solutions.

## Supplementary Information

Supplementary Information.
