# Supplementary material for: Development of highly sensitive metal-ion chemosensor and key-lock anticounterfeiting technology based on oxazolidine
Source: Sci Rep. 2022 Jan 20;12:1079. doi: 10.1038/s41598-022-05098-x (PMC8776736; doi:10.1038/s41598-022-05098-x)
Supplement: Supplementary file 1 — Supplementary Information. [file 41598_2022_5098_MOESM1_ESM.docx]

**Highly sensitive metal-ion chemosensors based on oxazolidine for encryption and anticounterfeiting: printing of key-lock security marks by ionochromic cellulosic papers**

***Bahareh Razavi,^1^ Hossein Roghani-Mamaqani^1,2*^ Mehdi Salami-Kalajahi^1,2^***

^1^ Faculty of Polymer Engineering, Sahand University of Technology, P.O. Box 51335-1996, Tabriz, Iran

^2^ Institute of Polymeric Materials, Sahand University of Technology, P.O. Box 51335-1996, Tabriz, Iran

* Corresponding author: Tel/Fax: +98 413 3459104. E-mail address: r.mamaghani@sut.ac.ir (H. Roghani-Mamaqani)

**SI. Experimental**

**SI1. ^1^H NMR analysis**

**
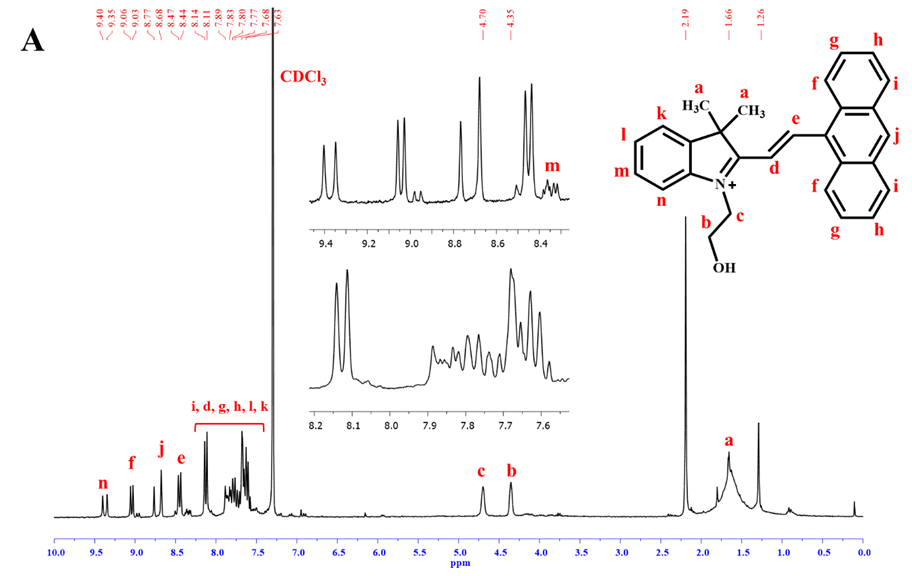
**

**
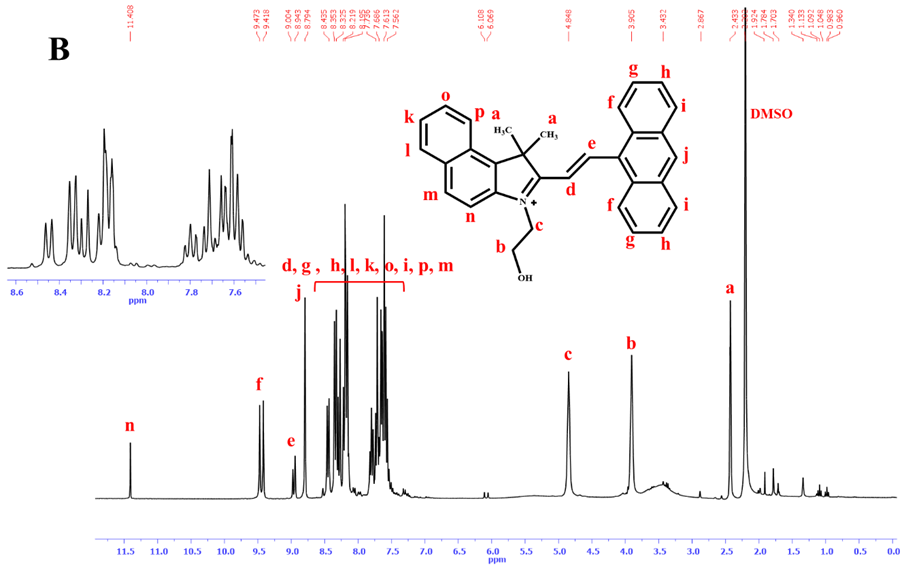
**

**
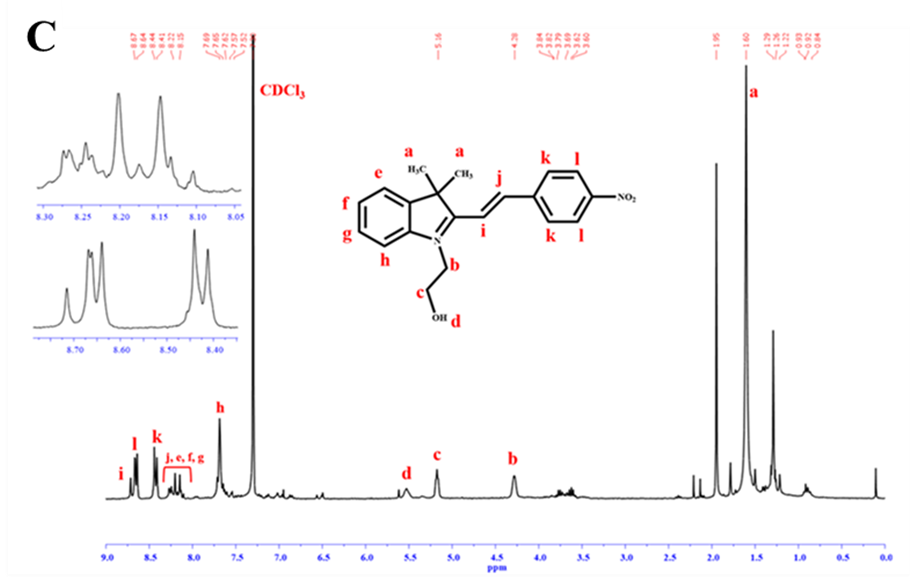
**

**
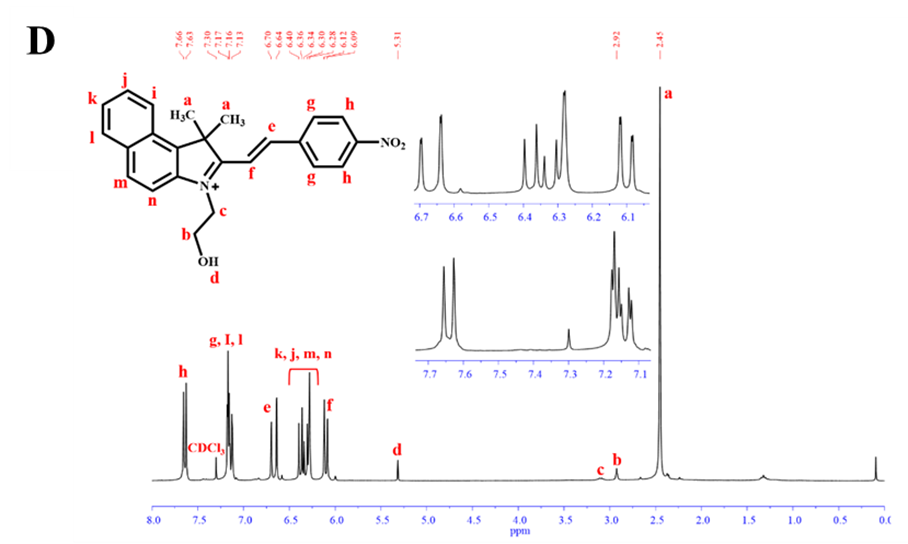
**

Figure S1- ^1^H NMR spectra of (A) OX­_1_-Anthracene, (B) OX­_2_-Anthracene (in DMSO), (C) OX_1_-Nitro, and (D) OX_2_-Nitro in CDCl_3_
